# Nurse Self-Evaluation of Assessment of Chemotherapy-Induced Peripheral Neuropathy in Patients With Cancer

**DOI:** 10.6004/jadpro.2012.3.5.5

**Published:** 2012-09-01

**Authors:** Constance Visovsky, Marilyn Haas, Beth Faiman, Sandra Kurtin, Anne Marie Shaftic, Elizabeth Lyden, Janique Rice

**Affiliations:** From University of South Florida College of Nursing, Tampa, Florida; CarePartners, Asheville, North Carolina; Cleveland Clinic Foundation, Cleveland, Ohio; University of Arizona Cancer Center, Tucson, Arizona; Holy Name Medical Center, Teaneck, New Jersey; University of Nebraska Medical Center, Omaha, Nebraska

## Abstract

The focus of this study was to assess the feasibility and clinical implementation of a standardized assessment for chemotherapy-induced peripheral neuropathy (CIPN) by registered nurses in patients undergoing neurotoxic chemotherapy. A total of 24 registered nurses from 4 different institutions were enrolled into the study. A pre- and posttest design was used to assess changes in nurses’ attitudes, knowledge, and perceived skill in CIPN assessment. Using selected data collection instruments, nurses performed standardized assessments during the course of chemotherapy treatments. Patient-reported symptoms, vibratory sensation, deep-tendon reflexes, and touch were collected at three time points during chemotherapy treatment. Results indicated there was no statistically significant change in knowledge of chemotherapy-induced peripheral neuropathy from baseline to the end of the study. However, this finding may be due to poor internal consistency noted among the items of the Nurse Knowledge and Attitudes CIPN Assessment. Implementation of a standardized subjective and objective nursing assessment of CIPN was feasible with a total mean feasibility score of 3.76 (range 0–5) with each individual item scoring between 3.35 and 3.91. The intervention did improve pretest and posttest confidence in performing assessment for CIPN (*p* = .003).

In the year 2011, approximately 207,090 new cases of invasive breast cancer, 142,570 cases of colorectal cancer, and 20,180 cases of multiple myeloma were predicted to be diagnosed in the United States (American Cancer Society, 2010). Treatment of these cancers requires the use of chemotherapeutic agents to effect cure or maintain disease control; however, cancer chemotherapy regimens with more intensive dosing schedules have induced significant neurotoxicity as the dose-limiting side effect.

Chemotherapy-induced peripheral neuropathy (CIPN) is the response of the peripheral nervous system to insult imposed following exposure to neurotoxic chemotherapy (Postma & Heimans, 2000). Sensory manifestations of CIPN include diminished proprioception, vibratory and cutaneous sensation and symptoms of numbness, tingling, burning, and pain. Motor neuropathy results in muscle atrophy and weakness. Autonomic symptoms such as urinary retention, constipation, alterations in blood pressure, and sexual dysfunction can be experienced. Difficulties with activities of daily living (ADL) such as buttoning clothing and writing have been reported (Verstappen, Heimans, Hoekman, & Postma, 2003; Gutiérrez-Gutiérrez, Sereno, Miralles, Casado-Sĸenz, & Gutiérrez-Rivas, 2010; Preston, 2000; Bakitas, 2007).

As novel therapies extend the lives of individuals affected by cancer, long-term functional deficits resulting from such treatments must now be addressed. Peripheral neuropathy has emerged as an important consequence of cancer therapy (Verstappen et al., 2003). Currently, there is no evidence-based, gold-standard assessment for CIPN. Nurses are on the front lines of patient-reported symptoms and objective assessment of clinical manifestations of CIPN. However, many nurses report that routine neuromuscular assessments for CIPN are not standardized in their clinical settings and that their institutions lack policies regarding assessment of CIPN.

## Literature Review

The sensitivity of the peripheral nervous system to toxic insult from chemotherapy is well established. Chemotherapy-induced peripheral neuropathy represents a twofold problem for patients. First, it is considered a dose-limiting side effect of therapy, resulting in chemotherapy dose reduction or cessation of treatment, potentially impacting drug efficacy and overall survival. Second, CIPN can significantly impair the patient’s quality of life due to neuropathic pain and/or functional limitations (Gutiérrez-Gutiérrez et al., 2010).

The addition of taxane preparations into chemotherapy regimens has increased the incidence of neurotoxicity, with 50% to 60% of all patients expected to develop CIPN. Taxanes can induce sensory and motor peripheral neuropathy by impairing axon structure and function (Partridge & Winer, 2004; Eniu, Palmieri, & Perez, 2005; Kuroi & Shimozuma, 2004; Vaishampayan, Parchment, Jasti, & Hussain, 1999).

Colorectal cancer is often treated with a platinum agent, often oxaliplatin. Oxaliplatin is known to induce two distinct types of peripheral sensory neuropathy: acute and chronic (delayed) neurotoxicity. The acute neurotoxicity is self-limiting and thus not a dose-limiting effect of oxaliplatin. The more chronic, cumulative neurotoxicity is correlated with the cumulative dose of oxaliplatin received. Unlike the acute effect, this chronic, sensory peripheral neuropathy is the dose-limiting toxicity associated with oxaliplatin administration and has been reported in 15% to 20% of patients in phase III clinical trials (Saif & Reardon, 2005; Grothey, 2005).

Bortezomib (Velcade), a proteasome inhibitor used in the first-line treatment of multiple myeloma, is known to induce significant peripheral neuropathy. The exact mechanism of bortezomib-induced peripheral neuropathy is unknown, but it is thought to be linked to metabolic changes in the dorsal root ganglia, mitochondrial dysregulation of calcium and neurotrophins (Argyriou, Iconomou, & Kalofonos, 2008).

Lenalidomide (Revlimid), is considered a specific therapeutic option for the treatment of refractory multiple myeloma when combined with dexamethasone (Cundari & Cavaletti, 2009). It has particular usefulness in patients pretreated with thalidomide (Thalomid) or bortezomib who have documented peripheral neuropathy from those agents. Thalidomide is also used in the treatment of refractory multiple myeloma and associated with the development of sensory peripheral neuropathy that may not be reversible, even when treatment ceases (Cundari & Cavaletti, 2009).

## Methods

**SETTING AND SAMPLE** 

This study used a pretest-posttest design enrolling 24 oncology nurses from 4 cancer institutions representing the midwestern, eastern, and southwestern United States who had previously participated in a CIPN educational program. Nurses who agreed to consider participation and who met eligibility criteria were referred to the principal investigator (PI), who provided each nurse participant with information about the study. Institutional review board approval was obtained at each institution, and informed consent was obtained from both nurse participants and the patients who agreed to be examined as part of the study. Oncology nurses were deemed eligible if they (a) were chemotherapy certified and (b) participated in a Web-based or regional CIPN educational program.

**PROCEDURES** 

Once recruited, the nurses in turn enrolled 52 patients for whom the planned treatment regimen included one or more of the following neurotoxic chemotherapy agent classifications: taxanes, platinums, proteasome inhibitors, thalidomide, and thalidomide derivatives.

**VARIABLES AND MEASURES** 

Outcome measures of interest were nurse attitudes and knowledge of CIPN, feasibility of implementing a standardized physical assessment of CIPN in a clinical setting, and the confidence and skill of the nurses performing the standardized assessments. Measures of nurses’ knowledge of and attitudes about CIPN were collected at baseline and at the end of the study using a PI-developed test: the Nurse Knowledge and Attitudes of CIPN Assessment. This assessment tool was based upon McCaffery and Ferrell’s Nurses’ Knowledge and Attitude Survey Regarding Pain (1997), a pretest and posttest evaluation measure for educational programs.

For the current study, the content validity for the Nurse Knowledge and Attitudes of CIPN Assessment was established by three CIPN expert reviewers. The instrument contains 30 items related to knowledge of CIPN, perception of CIPN assessment skill, intent regarding behavior change related to CIPN, and attitudes about CIPN training and assessment. Construct validity was examined during this study by comparing scores of nurses at various levels of educational preparation and oncology certification. Test-retest reliability and internal consistency were performed by repeated measures during this feasibility study.

Nurse self-assessment of confidence and skill in CIPN assessment was ascertained at baseline and at the end of the study period using the Confidence Scale (Grundy, 1993). The Confidence Scale comprises five items with responses that range from 1 (not certain at all) to 5 (absolutely certain for all steps), with higher scores indicating more confidence in one’s ability to perform the assessments. This scale was originally tested in both nursing students and registered nurses to determine their confidence level in performing physical assessment skills. The test-retest correlation ranged from .84 to .89, and Cronbach’s alpha for internal consistency for registered nurses was .84 (Grundy, 1993).

Data concerning the feasibility of implementing the standardized CIPN assessment in a busy oncology clinic setting were also collected at the end of the study period using a PI-developed Participant Evaluation of Feasibility and Acceptability Questionnaire. This instrument consisted of a seven-item Likert scale assessing content and applicability of the CIPN training and ability of incorporating CIPN assessment into clinical practice. Scores for the Likert-scale questions range from 1 (strongly disagree) to 5 (strongly agree) followed by 4 open-ended questions addressing the helpfulness, challenges, benefits, and suggestions for improvement in nurse training in CIPN assessment. A mean score of 3 or greater for the scaled items was considered indicative of intervention feasibility. In addition, a telephone conference focus group was held at each site with all participating nurses to elicit factors that were perceived as either barriers or facilitators to implementing standardized CIPN assessments over the study period.

**SELECTED PATIENT PROFILE** 

Each of the 24 registered nurses from the four institutions selected patients with diagnoses of breast cancer, multiple myeloma, and colorectal cancer who were scheduled to receive at least three cycles of neurotoxic chemotherapy with one of the selected agents as part of their treatment plan. Patients were assessed for symptoms and clinical manifestations of CIPN at three intervals: at baseline (before institution of chemotherapy) and at the next two scheduled cycles of chemotherapy. After informed consent was obtained, each cancer patient meeting the selected patient profile as noted above received the standardized set of CIPN assessments consisting of vibration sensation assessed by tuning fork at the great toe, deep-tendon reflexes tested at the right and left Achilles tendon, and touch sensation tested on the right and left hands and feet.

**DATA ANALYSIS** 

Descriptive statistics (mean, median, standard deviation [SD], and range) were used to describe the demographic characteristics of the nurse and patient sample. Variable distributions were examined using histograms, stem and leaf plots, measures of central tendency and dispersion, and frequency tables. A sample size of 24 nurses achieves 80% power to detect a difference of 0.6 SD, with a null hypothesis of a mean change of 0 (no difference between pre- and postassessment) at the .05 level of significance, using a two-sided Wilcoxon test to compare changes in nurses’ pre- and posttest knowledge dichotomized based upon degree or certification status.

Cronbach’s alpha was used to evaluate internal consistency of the Nurse Knowledge and Attitudes of CIPN Assessment. All quantitative analyses were completed using SAS Version 9.2. Lastly, content analysis was used to assess data from the telephone conference focus group with participating nurses at each site.

## Results

The descriptive characteristics of the participating nurses and patients can be found in Tables 1 and 2.

**Table 1:**
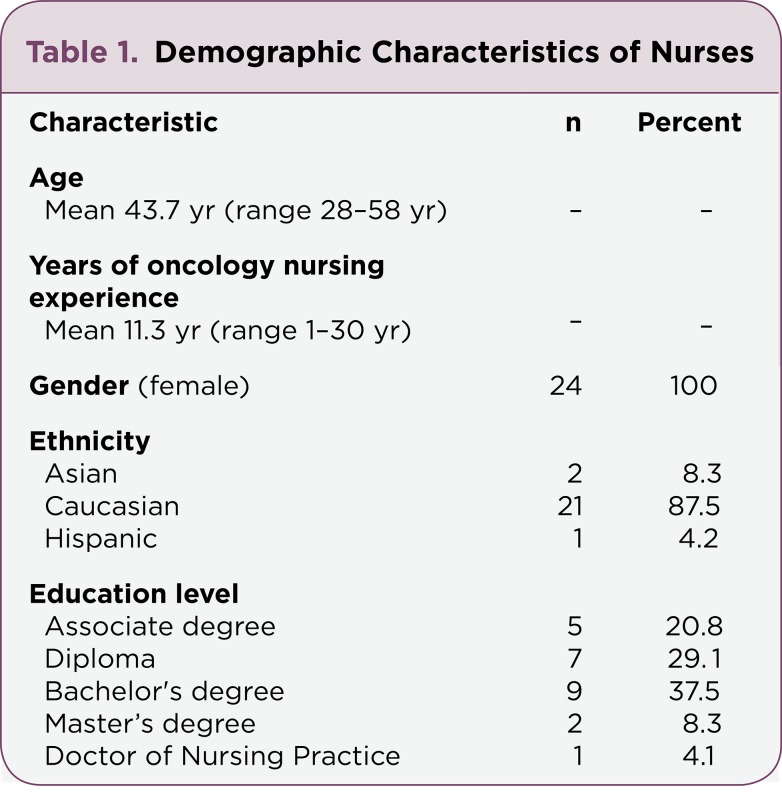
Table 1. Demographic Characteristics of Nurses

**Table 2:**
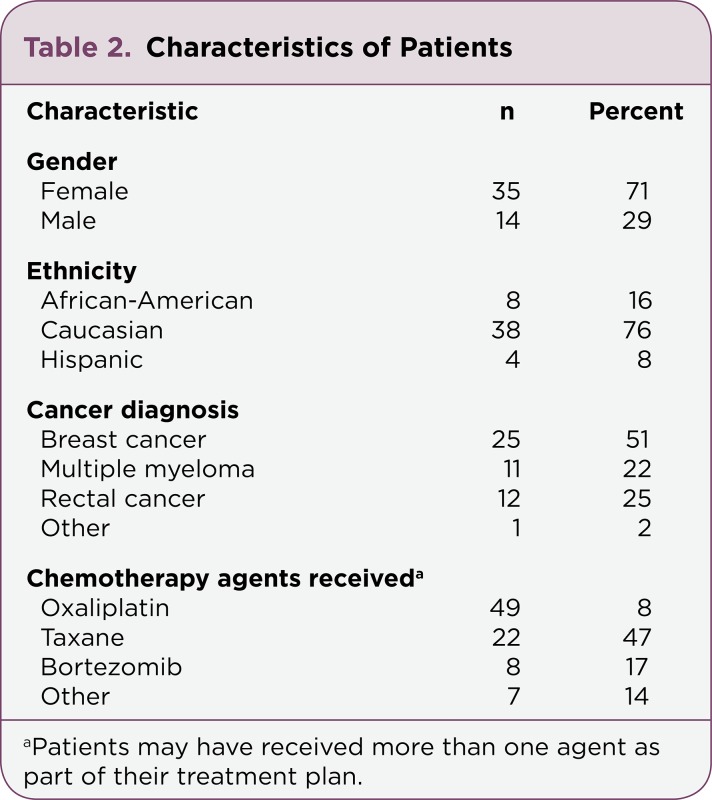
Table 2. Characteristics of Patients

**NURSE KNOWLEDGE AND ATTITUDES** 

The mean pretest score for Nurse Knowledge and Attitudes was 11 (SD = 2.14), with a range of 8 to 15 of a possible 30 questions answered correctly. The mean posttest scores remained unchanged. There were no statistically significant changes in knowledge of CIPN from pre- to posttest (*p* = .51). There were also no statistically significant differences in Nurse Knowledge and Attitudes between oncology-certified nurses (OCNs) and noncertified nurses, nor were there differences noted based on nursing degree (diploma, associate degree, bachelor’s degree, master’s degree, or DNP degree). Of note, there was poor internal consistency among the items of the Nurse Knowledge and Attitudes of CIPN Assessment (Cronbach’s alpha: 0.41 pretest, 0.447 posttest).

**FEASIBILITY AND ACCEPTABILITY** 

Each of the seven items of the Likert-scale portion of the Participant Evaluation of Feasibility and Acceptability Questionnaire scored above the 3.0 threshold for feasibility and acceptability of the CIPN assessment. Mean scores for the specific items ranged from 3.35 to 3.91, with an overall mean score for the instrument of 3.76. Table 3 contains the itemized analysis by each question.

**Table 3:**
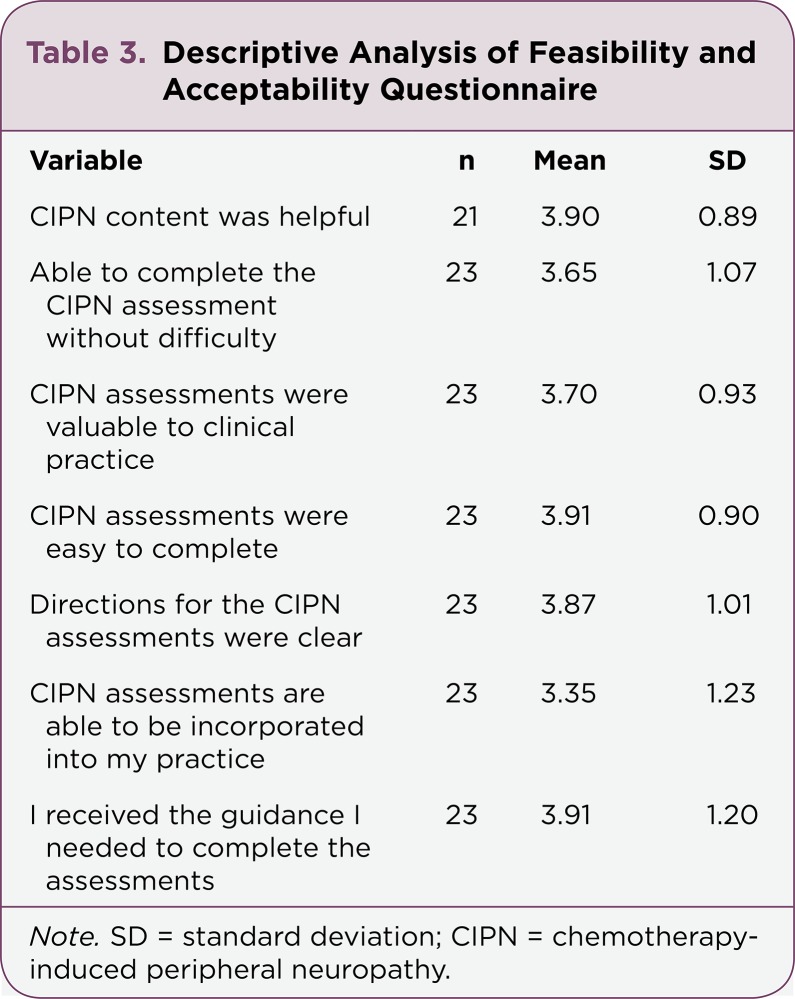
Table 3. Descriptive Analysis of Feasibility and Acceptability Questionnaire

Responses to the four open-ended questions indicate that 21.7% (n = 5) of the nurses found the study handouts outlining the step-by-step assessment instructions to be most helpful in assisting them in performing the standardized CIPN assessments. Thirteen percent (n = 3) of the nurses cited the ease of use of the assessment tools and changes observed in their personal assessment performance as being most helpful, while 8.7% (n = 2) of the nurses reported the knowledge gained to be most helpful in performing the CIPN assessments. A lack of time to perform the standardized assessments was reported by 26.1% (n = 6) of the nurses, and 17.4% (n = 4) reported difficulties with scheduling of patients within the nurse’s care assignment as being the greatest challenges preventing performance of the assessments. Knowledge concerning CIPN and appropriate evaluation techniques was cited by 56.5% (n = 13) as the benefits received by the participating nurses. Suggestions for other nurses who may be interested in conducting similar CIPN assessments were to practice the assessment skills and to develop a better understanding of CIPN and its effects on their patients.

At the end of the study, a telephone focus group meeting was held with nurses from each of the four sites to determine the factors perceived as either barriers or facilitators to implementing the standardized CIPN assessments over the study period. Results indicated that the educational aspects of the study such as assessment, hard copies of the assessment procedures, live demonstrations, and practicing the technique associated with certain physical assessments (Achilles tendon reflex) were the most helpful in assisting nurses to perform the assessments. The greatest challenge to performing the CIPN assessments cited by nurses at all four sites was limited time. Logistical challenges, such as varied patient treatment and nurse work schedules, also posed challenges. At times, the ability to elicit the Achilles tendon reflex posed difficulties in determining the accuracy of the reflex response.

Nurses cited several benefits from study participation, such as increased knowledge of CIPN and confidence in physical assessment skill to more accurately evaluate CIPN. Suggestions for other nurses who would like to incorporate these CIPN assessments into their practice included a quick pictorial guide to serve as a reference guide for the physical examination aspects of the assessment, offering education on CIPN for nurses, and considering adding activities to support learning.

**CONFIDENCE AND SKILL** 

The mean pretest confidence scale score was 17.6 (SD = 5.28) and the posttest score was 20.74 (SD = 2.78), with posttest scores of confidence and skill increasing an average of 3.8 points. There was a significant difference (*p* = .0037) in pretest and posttest scores of nurses in confidence and skill for performing the subjective and objective assessments of CIPN in patients.

## Discussion

Currently, there are no clinical guidelines to determine which neurologic physical examination components should be included in an assessment for CIPN. Guidelines as to when and how often patients receiving chemotherapy should be monitored for CIPN are also lacking. While a variety of instruments are available for assessment of CIPN, the most commonly used instrument is the National Cancer Institute’s Common Terminology Criteria for Adverse Events (CTCAE; National Cancer Institute, 2006). These criteria have been used to assess treatment toxicity and guide treatment delays and/or reductions, rather than as a gold standard for CIPN assessment.

There is a decided lack of research related to the neurologic assessment of CIPN by oncology nurses who administer chemotherapy commonly associated with neurotoxicity and advanced practice nurses (APNs) in the outpatient setting. Oncology nurses and APNs working in outpatient settings are positioned to be the first line for CIPN assessment. Patient interviews, including a review of symptoms and the impact on daily activities, together with observation of aspects of physical function such as gait and balance, are core components of each clinic visit. To the authors’ knowledge, no studies have been conducted to examine chemotherapy and APN knowledge of CIPN; the feasibility of conducting a more thorough, standardized CIPN assessment; and nurses’ confidence and skill in performing such assessments.

Knowledge of CIPN did not appear to be influenced by participation in a prior CIPN educational program. The varying length of time from the educational program to the start of the study may have been too lengthy to allow retention of the material. While our study found no significant change in knowledge and attitudes of CIPN over the course of the study, the nurse’s self-reported increased knowledge in both the open-ended questions of the Participant Evaluation of Feasibility and Acceptability Questionnaire, and perceived increased knowledge was a benefit the nurses reported in the telephone focus groups. Although the Nurse Knowledge and Attitudes of CIPN Assessment test was reviewed by three health-care professionals with expertise in CIPN, the item’s poor internal consistency may indicate that it failed to measure knowledge gained from the CIPN educational program nurses attended prior to the study. In addition, the relatively small sample of 24 nurses may not have yielded adequate power to detect differences in the pretest and posttest.

Study results indicate that despite the challenges of busy oncology practices, nurses felt it was indeed feasible and acceptable to conduct the assessments in the course of their practice. The use of handouts, a photo guide, and step-by-step examination instructions, in addition to reinforced physical assessment practice sessions, contributed to the feasibility of conducting the standard assessments. Nurses were trained in the physical examination by the oncology APNs at each site and had opportunities to have repeated reinforcement of the examination technique and sequence. Nurses reported an ability to see demonstrated improvements in their performance of the assessments over time. Challenges such as having enough uninterrupted time to perform the assessments, and the ability to schedule specific patients with the same nurse to perform the three repeated assessments, also posed difficulties.

The telephone focus groups held with nurses at each study site echoed a similar lack of time and difficulty with schedules and logistics for performing the same assessments on the same group of patients for each individual nurse. While the nurses liked the study materials, having a shorter "quick" or pocket guide for the physical assessment portion of the exam may have been more convenient. Another suggestion was to incorporate more ADL tasks, such as writing or buttoning clothing, into the standardized assessment and to use them as indicators of change in CIPN. This was not a feasible option for the purposes of this research, due to the difficulties in determining a methodologically sound means of evaluating change in handwriting or buttoning over time.

There was a significant increase in nurse-reported confidence and skill in performing the assessments over the course of the study period, with an average score increase of 3.8 points from pretest to posttest. Strengths of the study are the battery of standardized neurologic assessment of CIPN and the repeated measures design.

Only one other study of oncology nurse assessment of CIPN could be found. Binner, Ross, and Browner (2011) conducted a cross-sectional study to explore the practice behaviors and knowledge of 39 oncology nurses of CIPN in two hospital outpatient chemotherapy clinics. A PI-developed questionnaire containing 16 practice behavior items, 16 knowledge items, 8 instruction and perception items, and a 9-item demographic survey was used. Study results indicated that nurses had deficits in knowledge of CIPN and lacked training, proficiency, and confidence regarding neurologic physical assessment. Results of the present study support these findings.

While no differences in knowledge and attitudes of CIPN based upon nursing degree were found in this study, it is important to note that comprehensive neurologic examination may not be part of a nurse generalist education at the associate degree or diploma levels. Nurses educated at either the associate degree or diploma level may not have had a comprehensive physical examination course as part of their nursing education program. Of note, neurologic assessments that could be incorporated into a standardized physical examination for CIPN are not commonly performed by clinical nurses or APNs in oncology settings with the exception of neuro-oncology (Armstrong, Almadrones, & Gilbert, 2005). Thus, these assessments must be reviewed and practiced to obtain proficiency.

## Limitations

Nurses in the present study represent various regions of the country and both academic medical centers and a community oncology practice. Even so, the small sample size may not be representative of oncology nurses nationwide. Additionally, while missing data were not excessive, unanswered questions to the pre- and posttest nurse measures of CIPN knowledge and attitudes could have impacted study results in this small sample size.

## Conclusions

There is a lack of research regarding real-time assessment of CIPN using well-known clinical instruments by either bedside nurses or advanced practitioners. Additional research is necessary using neurologic physical assessment data in studies of CIPN. Such data are necessary to establish the most feasible, accurate means of detecting and monitoring CIPN and to establish clinical practice guidelines for CIPN assessment. Oncology advanced practitioners should not overlook the need for education and training regarding CIPN when considering the use of standardized assessments for research or clinical practice purposes in the patient care setting.
